# Acceptability of rectal self-sampling in non-clinical venues for chlamydia and gonorrhea testing among men who have sex with men: A cross-sectional study in Shenzhen, China

**DOI:** 10.3389/fpubh.2022.992773

**Published:** 2022-11-17

**Authors:** Rongxing Weng, Ning Ning, Chunlai Zhang, Lizhang Wen, Jianbin Ye, Honglin Wang, Jing Li, Xiangsheng Chen, Yumao Cai

**Affiliations:** ^1^Department of STD Control and Prevention, Shenzhen Center for Chronic Disease Control, Shenzhen, China; ^2^Shantou University Medical College, Shantou University, Shantou, China; ^3^Chinese Academy of Medical Sciences and Peking Union Medical College Institute of Dermatology, Nanjing, China; ^4^National Center for STD Control, China Center for Disease Control and Prevention, Nanjing, China

**Keywords:** Chlamydia trachomatis (CT), Neisseria gonorrhoeae (NG), men who have sex with men–MSM, rectal self-sampling, non-clinical venues

## Abstract

**Background:**

Rectal Chlamydia trachomatis (CT) and Neisseria gonorrhoeae (NG) infections among men who have sex with men (MSM) have become an increasingly important concern. The study aimed to explore (1) the acceptability of rectal self-sampling for chlamydia and gonorrhea testing among MSM in non-clinical venues in Shenzhen city, China; (2) factors associated with the acceptability of rectal self-sampling; and (3) factors associated with rectal CT and NG infections, respectively.

**Methods:**

This cross-sectional study was conducted in two non-clinical settings in Shenzhen, China, from April 2021 to October 2021. Mixed-effects logistic regression analysis was performed to explore the factors associated with acceptance of rectal self-collection for CT and NG testing.

**Results:**

Of the 306 MSM who were offered to perform rectal self-sampling, 133 (43.46%) accepted, and 96.24% (128/133) of them successfully provided a valid rectal sample. The prevalence of urogenital CT and NG infections among 303 MSM was 4.29 and 0.66%, respectively. The prevalence of rectal CT and NG infections among 128 participants was 31.25 and 9.38%, respectively. Participants having been diagnosed with HIV infection showed a higher acceptance of rectal self-collection for CT and NG testing.

**Conclusion:**

This study reported that rectal self-sampling in non-clinical venues for CT and NG testing among MSM was barely acceptable and feasible in China. Most CT and NG infections would have been missed if urethral screening was offered alone, which implies that the CT and NG screening should be scaled up in the above setting. Integrating free CT tests into regular STI interventions for MSM could also be considered.

## Introduction

Chlamydia trachomatis (CT) and Neisseria gonorrhoeae (NG) infections are common curable sexually transmitted infections (STI). It is estimated that, in 2016, the global prevalence of CT and NG in 15–49-year-old men was 2.7 and 0.7%, respectively (1). Untreated CT and NG could lead to serious complications in men including epididymitis, reactive arthritis, mucosal inflammation in the oral and anorectal areas, anorectal pain, discharge, and severe scarring (mostly related to the presence of genotype L of CT) (2, 3), and also increase the risk of the human immunodeficiency virus (HIV) transmission (4). For men, around 50% of CT cases and 10% of NG cases were asymptomatic in urethral samples, which indicates the importance of CT and NG screening (5, 6).

Compared with the general population, a higher prevalence of CT and NG infections among men who have sex with men (MSM) was found in many studies (2, 7). Extragenital CT and NG infections among MSM have become an increasingly important concern as extragenital-only infections are very common. More than 85% of extragenital CT infections and 70% of NG infections would have been missed if only urogenital tests were offered (7). Also, pharyngeal and rectal CT and NG infections are more likely to be asymptomatic compared with genital infections (8). Routine screening of extragenital sites among MSM was recommended in several countries (9, 10).

The self-sampling method has been increasingly used as it is preferable, acceptable (11), highly sensitive, and comparable with clinician collection (12), and because physical distancing was suggested in the pandemic of COVID-19. To reach out to more MSM such as those without being regularly seen by a clinician, self-sampling in non-clinical venues including home or non-government organizations (NGO) may be an option or an intervention strategy. There were no studies reporting self-sampling in non-clinical settings in China, and previous studies were only conducted in clinical settings with clinician-sampling (13, 14). Also, there were no policies or strategies to guide extragenital CT or NG screening among MSM in China. Understanding the acceptability of rectal self-collection among MSM in non-clinical venues in the Chinese context could inform the health authorities in developing related policies or strategies. The current study, as a part of the Shenzhen Gonorrhea and Chlamydia Intervention Programme (SGCIP), aimed to explore (1) the acceptability of rectal self-sampling for chlamydia and gonorrhea testing among MSM in non-clinical venues in Shenzhen city, China; (2) factors associated with the acceptability of rectal self-sampling; and (3) factors associated with rectal CT or NG infections.

## Materials and methods

### Study setting and sampling

This cross-sectional study was conducted in two non-clinical settings in Shenzhen city, China. One setting (Site 1) is a local NGO serving an urban, districtwide catchment area in Luohu district, which provided free HIV/STIs testing and counseling for MSM in collaboration with Shenzhen Center for Disease Control and Shenzhen Center for Chronic Disease Control. Another setting (Site 2) is a local rainbow counseling center serving an urban, citywide catchment area, which was one of the largest centers in Shenzhen to provide free HIV/STIs testing, counseling, and referral for MSM. MSM who seek an HIV/STI checkup or counseling services are encouraged to access these two settings through both booking and walking in. Partner notification is also used to expand services to more MSM. In the current study, the convenience sampling method was used to recruit MSM. The inclusion criteria were: (1) age ≥ 18 years; (2) willing to participate and cooperate in the study and provide informed consent; (3) men who had anal or oral sex with another man in the last 12 months; (4) without presenting any CT/NG related symptom. Individuals were only eligible to enroll once in the study to avoid duplicate individuals in the analysis. To ensure confidentiality, participants' questionnaires, biological samples, and CT and NG test results were anonymized by assigning unique survey identification numbers. From April 2021 to October 2021, after signing a written informed consent, all eligible attendees in the study site were invited to fill out a questionnaire and were offered to provide urine and self-collected rectal swabs regardless of their self-reported exposure site(s). All participants provided written informed consent. This study was approved by the Ethical Review Committee of the Shenzhen Center for Chronic Disease Control (Approval No.20180206).

#### Sample size calculation

As the current study was a pilot study to implement rectal self-sampling, the lowest acceptance rate in previous studies was selected (15). Setting *P* = 0.35 (self-sampling acceptance rate), d = 0.2*P* (allowable error), a = 0.05, Z_a_ = 1.96, the number of sample size was calculated according to the formula, $N=\frac{Z_{a}^{2}*p\left(1-p\right)}{d^{2}}$, and the result was N1 = 179. However, considering the possibility of incomplete information due to errors from sample or questionnaire collection, we increased this sample size by 20% based on N1 and the final sample size was 215.

### Questionnaire

For those who were eligible and provided informed consent, a structured paper-based questionnaire was used to obtain data on sociodemographic characteristics (e.g., age, marital status, and education), sexual behaviors, CT and NG infections, testing history, and opinions on CT testing and partner notification. Education was divided into two levels: (1) High school and below, including without any education, primary school, middle school, and high school, and (2) Junior college or higher, including junior college, bachelor's degree, master's degree, and doctoral degree. Information about sexual behaviors was collected referring to a standardized measure of the STI surveillance questionnaire in China.

### Specimen collection and laboratory testing

All respondents were provided a diagram and oral instructions by trained staff in each study site about how to perform rectal self-collection and all specimens were collected at visit. DNA was extracted and purified from the specimens by automated magnetism nucleic acid isolation method using the MagNA Pure 96 System (Roche, Switzerland) according to the manufacturer's instructions. Polymerase chain reaction (PCR) of Cobas 4800^®^ System (Roche, Switzerland) was used with Cobas^®^ 4800 CT/NG Amplification/Detection Kit to determine CT and NG infection. Participants were informed of positive laboratory results by phone for further intervention as soon as the results were available.

### Statistical analysis

All data from the questionnaires and laboratory results were double entered into computer using Epi Data software (Epi Data for Windows; The Epi Data Association Odense, Denmark) to establish a dataset. Frequencies (%) were used in categorical variables and mean ± SD was used in continuous variables. The **χ^2^** test was used to explore the differences of acceptance of rectal self-collection in the categorical variables. Also, the **χ^2^** test was used to explore the differences in the categorical variables between respondents with anorectal infection of CT/NG and respondents without anorectal infection of CT/NG. The acceptance of rectal self-collection was defined as a dependent variable for a mixed-effects logistic regression model, while age, marital status, Shenzhen residency, length of residency, education, ways to find sexual partners, previous HIV infection, and CT/NG testing history were included as fixed effects (Inclusion criteria: *P* < 0.10 in the **χ^2^** test) and study site was included as a random effect. The rectal NG infection was defined as a dependent variable for a mixed-effects logistic regression model, while Shenzhen residency and condom use during last anal sex were included as fixed effects (Inclusion criteria: *P* < 0.10 in the **χ^2^** test), and study site was included as a random effect. All data analysis was performed with the R program (Version 4.1.1). All tests were two-tailed, and *P* < 0.05 was defined as statistical significance.

## Results

### Acceptance of rectal self-sampling and baseline characteristics

The number of total samples was 306, and 183 MSM and 123 MSM were enrolled in site 1 and site 2, respectively. As shown in Table 1, around half of the participants were younger than 30 years old (48.37%, 148/306) and about one-fifth (21.57%, 66/306) were married. Around three-fifths (63.07%, 193/306) received Junior college or higher education, and 76.80% (235/306) had lived in Shenzhen for 2 years or more. Around one-fifth (19.05%, 56/294) of participants had been diagnosed with HIV infection.

**Table 1:** Characteristics, sexual behaviors, and CT/NG-related information of 306 men who have sex with men according to rectal self-collection acceptance among two non-government organizations in Shenzhen.

**Variables**	**Overall**	**Accepted**	**(%)**	**Refused**	**(%)**	***χ^2^***	***P***	**AOR**	***P***
	**N**							**(95%CI)**	
Total	306	133	(43.46)	173	(56.54)				
Age groups (*n* = 306)						3.69	0.05^*^		
≥ 30	158	77	(48.73)	81	(51.27)			1	
< 30	148	56	(37.84)	92	(62.16)			1.59 (0.75–3.36)	0.23
Marital status (*n =* 306)						8.36	< 0.01^*^		
Married	66	39	(59.09)	27	(40.91)			1	
Single/Divorced/Widowed	240	94	(39.17)	146	(60.83)			0.98 (0.41–2.34)	0.97
Registered residence in Shenzhen (*n =* 306)						4.25	0.04^*^		
Yes	154	58	(37.66)	96	(62.34)			1	
No	152	75	(49.34)	77	(50.66)			1.00 (0.50–2.00)	0.99
Length of residency (*n =* 306)						4.61	0.03^*^		
< 2year	71	23	(32.39)	48	(67.61)			1	
≥2year	235	110	(46.81)	125	(53.19)			1.29 (0.58–2.87)	0.54
Education level (*n =* 306)						18.27	< 0.01^*^		
High school or below	113	67	(59.29)	46	(40.71)			1.90 (0.91–3.99)	0.09
Junior college or higher	193	66	(34.20)	127	(65.80)			1	
Sexual orientation (*n =* 306)						0.09	0.76		
Heterosexuality	87	39	(44.83)	48	(55.17)				
Homosexuality/bisexuality	219	94	(42.92)	125	(57.08)				
Ways to find sexual partners (*n =* 301)						11.63	< 0.01^*^		
Others	32	23	(71.88)	9	(28.13)			1	
Bars/ parks	14	7	(50.00)	7	(50.00)			1.50 (0.22–10.39)	0.68
Hookup websites or geosocial networking applications	255	103	(40.39)	152	(59.61)			1.11 (0.35–3.53)	0.87
Rectal sexual behaviors in the past six months (*n =* 306)						0.86	0.35		
Yes	253	113	(44.66)	140	(55.34)				
No	53	20	(37.74)	33	(62.26)				
Condom use during last anal sex (*n =* 253)						4.03	0.04^*^		
Yes	190	78	(41.05)	112	(58.95)			1	
No	63	35	(55.56)	28	(44.44)			1.88 (0.93–3.83)	0.08
Condom use during anal sex in the past 6 months (*n =* 253)						0.10	0.75		
Inconsistent	117	51	(43.59)	66	(56.41)				
Consistent	136	62	(45.59)	74	(54.41)				
Having casual sexual partners in the past six months (*n =* 277)						0.09	0.76		
Yes	155	74	(47.74)	81	(52.26)				
No	122	56	(45.90)	66	(54.10)				
Previous HIV infection (*n =* 294)						21.61	< 0.01^*^		
No	191	78	(40.84)	113	(59.16)			1	
Yes	56	37	(66.07)	19	(33.93)			3.01 (1.32–6.88)	< 0.01^*^
Never tested	47	10	(21.28)	37	(78.72)			0.76 (0.27–2.15)	0.60
Ever CT/NG tested (*n =* 306)						12.25	< 0.01^*^		
No	254	99	(38.98)	155	(61.02)			1	
Yes	52	34	(65.38)	18	(34.62)			1.46 (0.62–3.43)	0.39
Urine CT (*n =* 303)						0.13	0.72		
Positive	13	5	(38.46)	8	(61.53)				
Negative	290	126	(43.45)	164	(56.55)				
Urine NG (*n =* 303)									
Positive	2	1	(50.00)	1	(50.00)	0.04	0.85		
Negative	301	130	(43.19)	171	(56.81)				

A mixed–effects logistic regression model with study site as a random effect was used for estimating the parameters.

* *P* < 0.05.

AOR, adjusted odds ratio; CI, confidence interval; CT, chlamydia trachomatis; NG, Neisseria gonorrhoeae.

All participants provided a urine sample with three invalid samples (3/306) due to an insufficient amount of urine. Around two-fifths (43.46%, 133/306) accepted to perform rectal self-sampling. Only 3.00% (4/133) of participants failed to collect and provide a rectal sample, and the reasons for failure to rectal self-sampling were rectal bleeding (2 participants) and difficulty in the collection (2 participants). Among those who refused to perform rectal self-sampling, only 8.67% (15/173) of them reported the reasons for the refusal including perceived no risk of rectal infection (13 participants) and feeling uncomfortable (2 participants).

### CT/NG infections and testing history of participants

The prevalence of urogenital CT and NG infections in 303 samples was 4.29 and 0.66%, respectively. There were no differences in the prevalence of CT and NG in urine samples between participants who accepted rectal self-sampling and those who did not accept (CT: χ^2^ = 0.13, *P* = 0.72; NG: χ^2^ < 0.01, *P* = 1.00). Among those who provided rectal self-sampling, almost all samples (99.22%, 128/129) were valid, except for one invalid sample with fecal contamination. The prevalence of rectal CT and NG infections among those who accepted rectal self-sampling was 31.25 and 9.38%, respectively. Both CT and NG infections of participants from rectal samples were significantly higher than those from urine samples (CT: χ^2^ = 41.23, *P* < 0.01; NG: χ^2^ = 19.06, *P* < 0.01). There were no differences in all variables between respondents with rectal infection of CT and respondents without rectal infection of CT (Table 2). Results from the mixed-effects logistic regression model suggested that there were no factors associated with rectal NG infection (Table 2). About CT/NG-related information (Table 1), only 16.99% (52/306) had been tested for CT or NG.

**Table 2:** Characteristics, sexual behaviors, and CT/NG–related information of 128 men who have sex with men according to rectal CT and NG prevalence among two non–government organizations in Shenzhen.

**Variables**	**Rectal CT N**	**(%)**	***χ^2^*** **values**	***p***	**Rectal NG N**	**(%)**	***χ^2^*** **values**	***p***	**AOR (95%CI)**	***p***
Age groups (*n =* 128)			0.49	0.49			< 0.01	0.92		
≥30	21	(28.77)			7	(9.59)				
< 30	19	(34.55)			5	(9.09)				
Marital status (*n =* 128)			2.84	0.09			0.04	0.85		
Married	7	(20.00)			3	(8.57)				
Single/Divorced/Widowed	33	(35.48)			9	(9.68)				
Registered residence in Shenzhen (*n =* 128)			0.48	0.49			4.16	0.04^*^		
Yes	16	(28.07)			2	(3.51)			1	
No	24	(33.80)			10	(14.08)			4.44 (0.83–23.69)	0.08
Length of residency (*n =* 128)			0.81	0.37			0.44	0.51		
< 2 year	9	(39.13)			3	(13.04)				
≥ 2 year	31	(29.52)			9	(8.57)				
Education level (*n =* 128)			1.98	0.16			1.34	0.25		
High school or below	24	(36.92)			8	(12.31)				
Junior college or higher	16	(25.40)			4	(6.35)				
Sexual orientation (*n =* 128)			1.16	0.28			0.10	0.75		
Heterosexuality	9	(24.32)			3	(8.11)				
Homosexuality/bisexuality	31	(34.07)			9	(9.89)				
Ways to find sexual partners (*n =* 128)			0.99	0.61			0.86	0.65		
Bars/ parks	1	(14.29)			1	(14.29)				
Hookup websites or geosocial networking applications	32	(32.32)			10	(10.10)				
Others	7	(31.82)			1	(4.55)				
Rectal sexual behaviors in the past six months (*n =* 128)			0.79	0.37			0.07	0.79		
Yes	36	(32.73)			10	(9.09)				
No	4	(22.22)			2	(11.11)				
Condom use during anal sex in the past 6 months (*n =* 110)			0.88	0.35			0.06	0.81		
Consistent	18	(37.50)			6	(9.68)				
Inconsistent	18	(29.03)			4	(8.33)				
Condom use during last anal sex (*n =* 110)			0.06	0.81			4.51	0.03^*^		
Yes	25	(32.05)			10	(12.82)			1	
No	11	(34.38)			0	(0.00)			< 0.001	0.97
									(< 0.001–>999.999)	
Having casual sexual partners in the past six months (*n =* 125)			0.35	0.55			0.47	0.50		
Yes	24	(27.45)			6	(8.11)				
No	14	(32.43)			6	(11.76)				
HIV infection (*n =* 120)			2.58	0.27			0.06	0.97		
No	19	(25.68)			7	(9.46)				
Yes	15	(40.54)			4	(10.81)				
Never tested	3	(33.33)			1	(11.11)				
Ever CT/NG tested (*n =* 128)			0.33	0.57			< 0.01	0.95		
No	31	(32.63)			9	(9.47)				
Yes	9	(27.27)			3	(9.09)				

A mixed–effects logistic regression model with study site as a random effect was used for estimating the parameters.

* *P* < 0.05.

%, Constituent ratio.

AOR, adjusted odds ratio; CI, confidence interval; CT, chlamydia trachomatis; NG, Neisseria gonorrhoeae.

### Factors associated with acceptance of CT and NG testing

As shown in Table 1, results from the mixed-effects logistic regression model suggested that participants having been diagnosed with HIV infection (AOR = 3.01, 95% CI = 1.32–6.88) were more likely to perform rectal self-collection for CT and NG testing (*P* < 0.05).

### Opinions to CT testing and partner notification

As shown in Table 3, more than half of the participants (58.88%) selected the routine CT test as the most appropriate time. Around one-third (35.53%) of respondents were willing to undertake a CT test if it is free, and half of them were willing to undertake a CT test without any condition. Most of them were willing to be engaged in patient-based partner referral if they were diagnosed with CT infection (Table 3).

**Table 3:** Opinion on CT testing and partner notification.

**Variables**	***N***	**(*%*)**
The most appropriate time to undertake a CT test (*n =* 304)		
Having CT–related symptoms	29	(9.54)
Routine CT screening (annual)	179	(58.88)
After risky sexual behaviors	41	(13.49)
Suggestion from doctors	55	(18.09)
Routine CT screening willingness (*n =* 304)		
Unwilling	15	(4.93)
Willing (If it is free)	108	(35.53)
Willing (If the participant has CT–related symptoms)	36	(11.84)
Willing (Without any condition)	145	(47.70)
Willingness to notify their partner(s) if they were diagnosed with CT infection (*n =* 303)		
No	25	(8.25)
Yes (provider referral)	49	(16.17)
Yes (patient–based partner referral)	210	(69.31)
Yes (expedited partner therapy)	19	(6.27)

%, Constituent ratio.

CT, chlamydia trachomatis.

## Discussion

To our knowledge, this is the first study in China to explore the acceptability of rectal self-sampling for chlamydia and gonorrhea testing among MSM in non-clinical venues. This study demonstrated that rectal self-sampling in non-clinical venues for chlamydia and gonorrhea testing among MSM was barely acceptable and feasible, with 43.46% (133/306) of MSM accepting rectal self-collection and 96.24% (128/133) of MSM successfully providing valid rectal samples in this pilot implementation. A previous study reported a similar acceptance rate (34.99%) of rectal self-sampling in community venues in Vancouver (15). A higher acceptance rate to perform self-collected rectal swabs was found in MSM attending STI clinics in The Netherlands (59.95%) and Ireland (91.55%) (16, 17). The potential reason why the acceptance rate in the study in Ireland was much higher than that in other studies is that the recruited population was HIV-positive MSM. The current study also showed that respondents who had been diagnosed with HIV infection were more likely to accept rectal self-collection, which was consistent with a previous study (15). Sanchez et al. found that HIV-positive MSM were more likely to report STI testing (18). Also, MSM living with HIV were more likely to have accessed NGO services, which further highlights the significance of promoting rectal self-sampling in NGO services (19). Besides, among those who refused rectal self-collection, a large proportion of them (86.67%, 13/15) perceived that they have no risk of rectal infection, which was consistent with the previous finding (20). The reasons for the refusal we found may provide a starting point for improving rectal self-sampling strategies among MSM in China. Information on the high prevalence of rectal CT/NG infection among MSM should be highlighted and delivered to all MSM for increasing the acceptance rate of rectal self-sampling.

The current study reached very high-risk MSM with a high prevalence of rectal CT and NG infections (31.25 and 9.38%, respectively), which was much higher than that (provider collection) in previous studies in China (CT: 11.23–15.57%, NG: 5.01–6.05%) (13, 14). Also, rectal CT infection in the current study was much higher than those MSM performing rectal self-collection in other studies (7.59–14.14%), which was not the case in the rectal NG infection (4.20–10.24%) (7, 8, 16, 21). In our study, among those who performed rectal self-collected swabs, 90.48% (38/42) of CT infection would have been missed and 92.31% (12/13) of NG infection would have been missed if urethral screening was offered alone, which highlighted the importance of rectal CT and NG screening outreach. In addition, there were no factors associated with the rectal CT and NG infection, which implies that all MSM should be screened with this high prevalence of rectal CT and NG infections. Future studies with a larger sample size could be considered to further support these findings.

The current study also found that just a small proportion of participants (16.99%) had had CT/NG test before, which was much lower than that in Australia (57.1%) (22). One important reason was that the first chlamydia screening program was launched in 2017 in China and a huge proportion of MSM was not tested before. Increasing the uptake of screening belongs to one of the major challenges to strengthen the continuum of STI prevention, diagnosis, treatment, and care (23). Previous studies reported an increase in acceptance rate over time after introducing self-taken extra-genital swabs (24, 25). Therefore, this acceptable rectal self-sampling strategy in non-clinical settings could help expand the uptake of CT/NG screening and detect more positive cases.

Our study also suggested that more than half of respondents believed that routine CT screening was the most appropriate to undertake CT test and most of them were willing to undertake routine CT screening. A previous study found that CT screening had great acceptance in this population (26). However, a third of participants were willing to undertake routine CT screening if the test is free, which suggested that offering free CT tests could expand the uptake of screening. Rectal CT/NG screening of MSM was proved to be a cost-effective, scalable intervention (27), which also supports the strategy of free CT tests for MSM. These findings implied that integrating free CT tests into regular STI interventions for MSM could be considered.

As a pilot implementation of rectal self-sampling in non-clinical settings, it indicated that rectal self-sampling was barely acceptable and the burden of rectal CT and NG among MSM was great, which suggested that integrating rectal self-sampling in non-clinical settings to STI services could be considered to expand surveillance efforts and reach populations who may be more resistant to clinic-based screening (20). Also, conducting self-sampling in non-clinical settings would not increase the work burden on healthcare providers (17). Future implementation should focus more on how to raise the acceptance rates of rectal self-sampling. Many reasons were reported such as fear of taking the swab incorrectly, finding instructions unclear, being unaware of their level of susceptibility to rectal infections, and believing urine testing would identify rectal infections (16, 20). Our findings also provided reasons for the refusal such as perceived no risk of rectal infection and feeling uncomfortable, which should be considered in the future implementation of this rectal self-sampling screening strategy. Novel methods such as postal self-sampling or Internet-based self-sampling could also be considered in the future.

Several limitations should be concerned. First, the convenience sampling method was used to recruit MSM in two non-clinical settings in Shenzhen city, China, which limit the representativeness to the MSM population in China and also may limit the generalizability of the results to other cities. However, over 70% of Shenzhen city's population are temporary migrants (28) and around half of the participants (49.67%, 152/306) belonged to the migrant population without registered residence in Shenzhen, which may represent the MSM population in China to some extent. Besides, MSM are usually hard to reach for research and two large non-clinical settings were included in the study to increase the possibility of generalizability. Second, information related to sexual behaviors was self-reported, which may lead to social desirability bias. Third, information related to NG testing and partner notification was not collected, which could be considered in the future implementation of the study.

In summary, this study found that rectal self-sampling in non-clinical venues for chlamydia and gonorrhea testing among MSM was barely acceptable and feasible. Most CT and NG infections would have been missed if urethral screening was offered alone, which implies that the CT screening should be scaled up in the above setting. HIV status should be taken into account to promote rectal screening.

## Data availability statement

The datasets presented in this article are not readily available because the dataset generated and analyzed during the current study could be available from the corresponding author on reasonable request. Requests to access the datasets should be directed to 64165469@qq.com.

## Ethics statement

The studies involving human participants were reviewed and approved by Ethical Review Committee of the Shenzhen Center for Chronic Disease Control. The patients/participants provided their written informed consent to participate in this study.

## Author contributions

RW and YC conceived and designed the study. NN, CZ, LW, JY, HW, and JL supervised the data collection. YC, XC, RW, CZ, LW, JY, HW, JL, and NN performed the research. RW, NN, and YC analyzed and interpreted the results and were the major contributors in writing the manuscript. XC, CZ, LW, JY, HW, and JL revised the manuscript critically. All authors read and approved the final manuscript.

## Funding

This work was supported by the Sanming project of Medicine in Shenzhen [grant number SZSM201611077].

## Conflict of interest

The authors declare that the research was conducted in the absence of any commercial or financial relationships that could be construed as a potential conflict of interest.

## Publisher's note

All claims expressed in this article are solely those of the authors and do not necessarily represent those of their affiliated organizations, or those of the publisher, the editors and the reviewers. Any product that may be evaluated in this article, or claim that may be made by its manufacturer, is not guaranteed or endorsed by the publisher.
